# COVID-19 in Primary and Secondary School Settings During the First Semester of School Reopening — Florida, August–December 2020

**DOI:** 10.15585/mmwr.mm7012e2

**Published:** 2021-03-26

**Authors:** Timothy Doyle, Katherine Kendrick, Thomas Troelstrup, Megan Gumke, Jerri Edwards, Shay Chapman, Randy Propper, Scott A. Rivkees, Carina Blackmore

**Affiliations:** ^1^Division of Disease Control and Protection, Florida Department of Health; ^2^Division of State and Local Readiness, Center for Preparedness and Response, CDC; ^3^Division of Community Health Promotion, Florida Department of Health; ^4^Florida Department of Health.

After detection of cases of COVID-19 in Florida in March 2020, the governor declared a state of emergency on March 9,* and all school districts in the state suspended in-person instruction by March 20. Most kindergarten through grade 12 (K–12) public and private schools in Florida reopened for in-person learning during August 2020, with varying options for remote learning offered by school districts. During August 10–December 21, 2020, a total of 63,654 COVID-19 cases were reported in school-aged children; an estimated 60% of these cases were not school-related. Fewer than 1% of registered students were identified as having school-related COVID-19 and <11% of K-12 schools reported outbreaks. District incidences among students correlated with the background disease incidence in the county; resumption of in-person education was not associated with a proportionate increase in COVID-19 among school-aged children. Higher rates among students were observed in smaller districts, districts without mandatory mask-use policies, and districts with a lower proportion of students participating in remote learning. These findings highlight the importance of implementing both community-level and school-based strategies to reduce the spread of COVID-19 and suggest that school reopening can be achieved without resulting in widespread illness among students in K–12 school settings.

Florida has one independent school district in each of its 67 counties. For the 2020–21 school year, 2,809,553 registered students were enrolled in approximately 6,800 public, charter, and private K–12 schools, ranging from 707 to 334,756 students per school district. In response to the COVID-19 pandemic, some school districts delayed the start of the 2020–21 academic year after suspension of in-person learning in March. Most schools resumed in-person instruction sometime during August 10–31, 2020, except those in the two largest school districts, Broward and Miami-Dade, which began remote learning in August but did not resume in-person instruction until October 9 and November 10, respectively. Statewide, as of September 24, 45% of registered students received full-time in-person instruction.

To assess the occurrence of COVID-19 in Florida schools after resumption of in-person instruction, CDC and the Florida Department of Health (FDOH) reviewed school-related cases and outbreaks during August–December 2020.^†^ County health department staff members conducted case investigations and contact tracing for all COVID-19 cases and reported data via the FDOH reportable disease surveillance system. A COVID-19 case was defined as nucleic acid amplification or antigen detection of acute infection with SARS-CoV-2 (the virus that causes COVID-19) in a symptomatic or asymptomatic person. A school-related case was defined as a COVID-19 case in a student or staff member who had been on campus for class, work, athletics, or other reasons during the 14 days preceding symptom onset or testing, and could reflect cases acquired in the school, home, or community setting. A school-based outbreak was defined as two or more epidemiologically linked school-related cases. Data regarding school start dates by district, student enrollment, and proportion of registered students receiving full-time in-person instruction were obtained from the Florida Department of Education. Information regarding temporary COVID-19–related school closures was obtained from FDOH staff members in the various counties. Data on school district mask use policies were obtained from reopening plans in each district (*1*). Descriptive statistics were computed; one-way analysis of variance and simple linear regression analyses were conducted to identify factors associated with student incidence by district. Statistical analyses were performed using JMP software (version 15.1; SAS Institute). This activity was reviewed by CDC and was conducted consistent with applicable federal law and CDC policy.^§^

During August 10–December 21, 2020, a total of 63,654 cases of COVID-19 among persons aged 5–17 years were reported to FDOH; during the same period, 34,959 school-related COVID-19 cases were reported, including 25,094 (72%) among students and 9,630 (28%) among staff members. Therefore, among all cases reported among school-aged children, 39.4% were classified as school-related (Figure). School-related cases in children occurred in <1% (25,094 of 2,809,553) of all registered students. Among all cases in children aged 5–17 years, the median age was 13 years (interquartile range = 9–15 years) and did not differ between cases that were and were not school-related. Among school-related cases, 101 hospitalizations and no deaths were reported among students, and 219 hospitalizations and 13 deaths were identified among school staff members. Among the 13 staff members who died, nine had risk factors for severe outcomes, including obesity (seven), age >60 years (four), and other chronic conditions (four); some reported probable exposures outside the school setting, including within the household.

**FIGURE:**
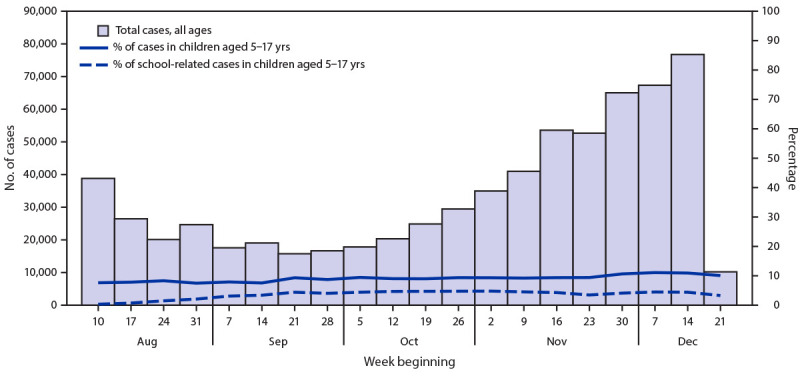
Weekly school-related COVID-19 cases reported among students, as a proportion of overall cases in children aged 5–17 years and in the general population — Florida, August–December 2020* * Week beginning December 21 is a partial week, only including December 21, 2020.

Contact tracing investigations identified 86,832 persons who had close school setting contact^¶^ with persons with cases of school-related COVID-19; among these, 37,548 (43%) received testing. Overall, 10,092 (27% of contacts who were tested) received a positive SARS-CoV-2 test result while in quarantine. Testing of symptomatic persons was encouraged; however, 11% of school contacts who had COVID-19–symptoms** were not tested.

A total of 695 school-based outbreaks were identified in 62 (93%) of 67 school districts, involving 4,370 total cases, for a statewide average of 6.3 COVID-19 cases per outbreak. Therefore, <11% (695 of 6800) of schools reported an outbreak. A subset of 562 (81%) outbreaks with additional information was further analyzed; 110 (20%) of these outbreaks were associated with activities outside the classroom setting, including sports (91), nonschool–sponsored social gatherings (12), or transportation to school (four). The most frequent extracurricular sports-related outbreaks involved football (27), basketball (14), volleyball (nine), wrestling (eight), dance (eight), cheerleading (seven), and soccer (six). Sports-related outbreaks were larger on average than were nonsports–related outbreaks (mean = 6.0 cases versus 4.1 cases; p<0.01). The four largest sports-related outbreaks involved two wrestling events (58 and 27 cases) and two football events (18 and 17 cases). Most sports-related outbreaks involved high school grade levels.

Through December 18, 2020, a total of 28 schools in 12 counties closed temporarily because of COVID-19, with a median closure duration of 4 days (range = 1–14 days); 16 (57%) closures occurred in public schools, nine (32%) in private schools, and three (11%) in charter schools. Partial closures of one or more classrooms, but not the entire school, occurred in 226 schools in 38 counties; 88% of these partial closures occurred in public schools, 8% in private, and 4% in charter schools. Elementary school grades accounted for 75% of partial closures.

Descriptive statistics for the 67 county-based school districts indicated that a median of 70% of students were attending school and receiving full-time in-person instruction as of September 24 (range = <1% [Miami-Dade and Broward] to 94% [Baker]) (Table 1). The median incidence among registered students was 1,280 per 100,000 students, ranging from 394 to 3,200 among counties.

**TABLE 1:** COVID-19 school-related cases in 67 county-based school districts — Florida, August 10–December 21, 2020

County characteristic	Median (range)
County population, all ages	130,642 (8,613–2,830,500)
Students enrolled in K–12 schools	15,306 (707–334,756)*
Students attending in-person full-time,^†^ median % (range)	70 (<1–94)
**COVID-19 incidence by county**	
County incidence^§^ in general population	3,163 (1,915–14,606)
Incidence of school-related student cases among all registered students^¶^	1,280 (394–3,200)
School-related cases among students	170 (18–2,780)
School-related cases among staff members	68 (9–863)
Ratio of student to staff member cases	2.5 (1.1–7.4)
No. of school-based outbreaks**	5 (1–69)
No. of cases associated with school-based outbreaks	31 (2–541)

**Abbreviation:** K–12 = kindergarten through grade 12.

* A total of 2,809,553 registered students were enrolled in approximately 6,800 public, charter, and private K–12 schools.

^†^ As reported by Florida Department of Education on September 24, 2020.

^§^ Total number of cases in the county during August 10–December 21, divided by county population, expressed per 100,000 persons.

^¶^ School-related cases in students by school district, during school start date and December 21, per 100,000 registered students (adjusted for school start date, i.e., adjusted rate = crude rate [131/x] where x = days from school start to December 18 and maximum number of days = 131).

** Two or more epidemiologically linked school-related cases.

Factors identified in bivariate analysis associated with student case rate by district were county population size, school opening during the first week, district reopening plans that included mandatory mask use, proportion of students attending in-person instruction, and the background case rate per county during August 10–December 21 (Table 2). Higher mean student case rates were reported from counties with the lowest population, districts opening school during August 10–14, and districts that did not mandate mask use in their reopening plans, compared with rates in larger counties, districts opening after August 16, and those with mask mandates. The background cumulative disease incidence during August 10–December 21 in each county was positively correlated with the incidence among students. The proportion of students, by district, attending full-time in-person instruction also positively correlated with the student case rate. In general, smaller counties resumed classes earlier, had a higher proportion of students attending in-person instruction, were less likely to mandate universal mask use in schools, and had higher student incidences (2,212 per 100,000 in the lowest county population quartile versus 970 in the highest).

**TABLE 2:** Factors associated with COVID-19 incidence — Florida, August 10**–**December 21, 2020

Factor	Student rate*	P-value
**County population size by quartile^†^**		
Q1: 8,613–28,089	2,212	<0.0001
Q2: 28,090–130,642	1,430	
Q3: 130,643–368,678	1,226	
Q4: 368,679–2,830,500	970	
**Opening date**		
August 10–14	1,882	0.01
After August 16	1,367	
**Masks mandated in district reopening plan** ^§^		
Yes	1,171	<0.01
No	1,667	
**Full-time in-person students^¶^**	R** = 0.5069	<0.0001
	R-squared = 0.2570	
**County case rate^††^**	R** = 0.4442	<0.001
	R-squared = 0.1973	

**Abbreviations:** Q = quartile; R = correlation coefficient.

* School-related cases in students by school district, during school start date and December 21, per 100,000 registered students (adjusted for school start date: adjusted rate = crude rate[131/x] where x = days from school start to December 18 and maximum number of days = 131).

^†^ Sixty-seven Florida counties divided into four groups (quartiles) with quartile 1 containing the 17 counties with the lowest population per county, and quartile 4 containing the 16 counties with the highest population per county. Each of the other quartiles contains 17 counties. County population range of each quartile is specified next to each quartile designation.

^§^ Twenty-seven (40%) school districts had reopening plans requiring masks in schools. Inclusion in plan might not be an accurate reflection of mask use in school setting.

^¶^ Proportion of students attending full-time in-person instruction (continuous 0%–100%).

** R is a measure of correlation between the continuous independent variable indicated in the Factor column and the continuous dependent variable of student case rate per 100,000. R-squared indicates the percent of variation in student case rate that is explained by the independent variable included in the regression model.

^††^ Per 100,000 population; excludes one outlier county (county with very small population and large outbreak in correctional facility, resulting in large county population rate with limited community spread).

## Discussion

Although COVID-19 can and does occur in school settings, the results of these analyses indicate that in Florida, 60% of COVID-19 cases in school-aged children were not school-related, <1% of registered students were identified as having school-related COVID-19, and <11% of K–12 schools reported outbreaks. These findings add to a growing body of evidence suggesting that COVID-19 transmission does not appear to be demonstrably more frequent in schools than in noneducational settings (*2*). Temporal trends in the United States also indicate that among school-aged children, school-based transmission might be no higher than transmission outside the school setting (*3*,*4*); the limited in-school transmission observed in Florida has also been observed in other states (*5*) and countries (*6*).

Success in preventing the introduction of SARS-CoV-2 into schools depends upon controlling community transmission and adhering to mitigation measures in schools, particularly masking, physical distancing, testing, and increasing room air ventilation (*2*,*4*,*7*). Where feasible, supporting family choice for remote versus in-person learning likely reduces in-school crowding and facilitates better physical distancing in schools. In Florida, a large proportion of school-related outbreaks was observed among social gatherings and extracurricular sporting activities. Household transmission and social gatherings might pose a higher risk for infection among school-aged children than does school attendance (*8*). School sports and other extracurricular activities in which masking and physical distancing are difficult or impossible to achieve should be postponed, particularly during periods of high community transmission (*2*,*9*).

The findings in this report are subject to at least six limitations. First, because data on the number of teachers and staff members statewide or by county were not available, rates of total school-related cases could not be calculated; instead, the number of student cases per 100,000 registered students was used. Second, screening testing was generally not done in most schools, therefore, asymptomatic infections might have been underascertained. Third, classification of school-related cases, contacts, and outbreaks was dependent on thorough case interviews and might have been incomplete, relative to the overall number of cases in school-aged children. Fourth, although the operational definition used for school-related cases was likely sensitive, it does not ensure that all persons with school-related cases acquired infection in the school setting because infections might have been acquired elsewhere. Fifth, limited data were available at the school district level on some mitigation measures, such as mask use in schools, so these mitigation measures could not be fully assessed. Finally, results should be interpreted with caution because most students in the largest school districts did not resume in-person education for the first part of the analysis period.

These findings provide further evidence that resumption of school can likely be achieved without the rapid disease spread observed in congregate living facilities or high-density worksites. Both community-level and school-based measures to prevent spread of disease are essential to reduce SARS-CoV-2 transmission in school settings (*10*).

**Summary**

**What is already known about this topic?**

Limited U.S. data have been reported regarding COVID-19 in students and school staff members as kindergarten through grade 12 (K–12) schools have reopened.

**What is added by this report?**

COVID-19 school-related disease incidence among Florida students was correlated with community incidence in the counties observed and was highest in smaller counties, districts without mask requirements, and those that reopened earliest after closure in March 2020. Incidence increased with the proportion of students receiving in-person instruction. Fewer than 1% of registered students were identified as having school-related COVID-19.

**What are the implications for public health practice?**

Both community-level and school-based mitigation measures are important in limiting transmission of COVID-19; school reopening can likely be achieved without widespread student illness in K–12 settings.

## Footnotes

*
https://www.flgov.com/wp-content/uploads/orders/2020/EO_20-52.pdf

† The last school day before Christmas break was December 18 in most districts; however, cases reported through December 21 were included to allow for testing and reporting time lag.

§ 45 C.F.R. part 46, 21 C.F.R. part 56; 42 U.S.C. Sect. 241(d); 5 U.S.C. Sect. 552a; 44 U.S.C. Sect. 3501 et seq.

¶ Close contact is defined as contact within 6 feet of a person with a case of COVID-19 for ≥15 minutes, within a 24-hour period.

**
https://www.cdc.gov/coronavirus/2019-ncov/symptoms-testing/symptoms.html
